# Value of IVIM in Differential Diagnoses between Benign and Malignant Solitary Lung Nodules and Masses: A Meta-analysis

**DOI:** 10.3389/fsurg.2022.817443

**Published:** 2022-06-01

**Authors:** Yirong Chen, Qijia Han, Zhiwei Huang, Mo Lyu, Zhu Ai, Yuying Liang, Haowen Yan, Mengzhu Wang, Zhiming Xiang

**Affiliations:** ^1^ Graduate School, Guangzhou University of Chinese Medicine, Guangzhou, China; ^2^ Department of Radiology, Guangzhou Panyu Central Hospital, Guangzhou, China; ^3^ School of Life Sciences, South China Normal University, Guangzhou, China; ^4^ Department of Oncology, Guangzhou Panyu Central Hospital, Guangzhou, China; ^5^ Department of MR Scientific Marketing, Siemens Healthineers, Guangzhou, China

**Keywords:** IVIM-DWI, magnetic resonance imaging, differential diagnosis, lung nodules, meta-analysis

## Abstract

**Purpose:**

This study aims to evaluate the accuracy of intravoxel incoherent motion diffusion-weighted imaging (IVIM-DWI) in distinguishing malignant and benign solitary pulmonary nodules and masses.

**Methods:**

Studies investigating the diagnostic accuracy of IVIM-DWI in lung lesions published through December 2020 were searched. The standardized mean differences (SMDs) of the apparent diffusion coefficient (ADC), tissue diffusivity (*D*), pseudo-diffusivity (D*), and perfusion fraction (*f*) were calculated. The sensitivity, specificity, area under the curve (AUC), publication bias, and heterogeneity were then summarized, and the source of heterogeneity and the reliability of combined results were explored by meta-regression and sensitivity analysis.

**Results:**

A total of 16 studies including 714 malignant and 355 benign lesions were included. Significantly lower ADC, *D*, and *f* values were found in malignant pulmonary lesions compared to those in benign lesions. The *D* value showed the best diagnostic performance (sensitivity = 0.90, specificity = 0.71, AUC = 0.91), followed by ADC (sensitivity = 0.84, specificity = 0.75, AUC = 0.88), *f* (sensitivity = 0.70, specificity = 0.62, AUC = 0.71), and D^*^ (sensitivity = 0.67, specificity = 0.61, AUC = 0.67). There was an inconspicuous publication bias in ADC, *D*, D* and *f* values, moderate heterogeneity in ADC, and high heterogeneity in *D*, D*, and *f* values. Subgroup analysis suggested that both ADC and *D* values had a significant higher sensitivity in “nodules or masses” than that in “nodules.”

**Conclusions:**

The parameters derived from IVIM-DWI, especially the *D* value, could further improve the differential diagnosis between malignant and benign solitary pulmonary nodules and masses.

**Systematic Review Registration:** https://www.crd.york.ac.uk/PROSPERO/#myprospero, identifier: CRD42021226664

## Introduction

Lung cancer is the leading cause of cancer-related deaths worldwide, with 1.8 million deaths (18%) reported in 2020 (1). Lung cancer has a poor prognosis; at the time of diagnosis, approximately 70% of patients are already at an advanced stage, and more than half of the people diagnosed with lung cancer die within one year of diagnosis. The 5-year survival is <18% (2, 3).

Early detection and characterization of solitary pulmonary lesions, especially the differentiation of benign and malignant pulmonary nodules, is important for risk assessment and management strategies. Low-dose CT (LDCT), which uses less radiation than a standard chest CT, has been proven effective in detecting early lung cancer and reducing mortality, especially among patients considered to be at high risk (4). Moreover, with the wide application of LDCT, an increase in the numbers of pulmonary nodules with unclear malignant tendencies has been observed, in turn affecting the treatment strategy (5, 6). Yet, the major limitations of the LDCT are (a) inability to differentiate benign from malignant pulmonary lesions (7, 8), (b) being unsuitable for long-term LDCT screening programs (due to cumulative radiation doses) (9), and (c) only suitable for certain patients (e.g., it is not recommended for pregnant women).

Diffusion-weighted imaging (DWI), a magnetic resonance imaging (MRI) method free from ionizing radiation and that requires no intravenous contrast agent, is based upon measuring the random Brownian motion of water molecules within a voxel of tissue, indicating changes at the cellular level (10). The apparent diffusion coefficient (ADC) value of DWI is usually lower in malignant lesions than that in benign lesions. However, ADC of conventional monoexponential DWI is not accurate enough to reflect the real diffusivity due to the influence of microcirculation (11, 12).

More recently, intravoxel incoherent motion (IVIM), proposed by Bihan et al. (13) in 1988 to distinguish the influence of the random microscopic motion of water molecules and the microcirculation of blood by applying a biexponential signal equation model, has been recently applied to distinguish benign and malignant pulmonary lesions, showing promising results. Nonetheless, the number of related studies is insufficient to provide faithful results, so its application is still debatable. Thus, the aim of this study was to systematically assess the diagnostic performance of IVIM-DWI in differentiating benign and malignant nodules and masses using meta-analysis.

## Methods

### Literature Search

Studies published through December 2020 in English or Chinese in PubMed, Web of Science, Cochrane Library, and China National Knowledge Infrastructure databases were searched. The following keywords were applied: (Lung Neoplasm OR Pulmonary Neoplasm OR Lung Cancer OR Pulmonary Cancer) AND (Intravoxel Incoherent Motion OR IVIM OR multiple *b*-value DWI OR biexponential). Reference lists of qualified studies were also manually searched.

### Study Selection

The following inclusion criteria were applied in study selection: (a) IVIM-DWI was used for differentiation of benign and malignant solitary pulmonary nodules and masses; (b) exploring the diagnostic performance of IVIM-DWI was the main purpose of the study; (c) the pathological evidence was used as diagnosis criteria; (d) the sensitivity and specificity about diagnostic performance were provided or enough information was reported to calculate the numbers of true-positive (TP), false-negative (FN), false-positive (FP), and true-negative (TN). The exclusion criteria were the following: (a) reviews, meta-analyses, conference abstracts, or dissertations; (b) duplication with the same study data from the same institutions; and (c) animal experiments.

### Data Extraction

The mean values and standard deviation (SD), sensitivity, specificity, threshold, and area under the curve (AUC), which presented the diagnostic performance of ADC, *D*, D*, and *f* values, were extracted. Other information, including the first author, year of publication, study design, number and age of patients, field strength and vendors, *b* values, repetition time, and echo time, were also analyzed. Data extraction was performed by one author and reviewed by another author. TP, FP, FN, and TN data were calculated when the numbers of malignant lung lesions and benign lung lesions and the sensitivity and specificity were provided.

### Quality Assessment

We assessed the quality of each included study using the Quality Assessment of Diagnostic Accuracy Studies-2 (QUADAS-2) (14), which includes four domains (patient selection, index test, reference standard, and flow and timing); each domain is answered with “yes,” “no,” or “unclear.” In our study, IVIM-DWI was designed as the index test and histopathologic confirmation as the reference standard. All assessment results were then imported into RevMan version 5.3 (The Nordic Cochrane Centre, Copenhagen, Denmark).

### Publication Bias and Heterogeneity Evaluation

Publication bias of continuous variables was assessed by Funnel plots, Begg’s test, and Egger’s test; publication bias of diagnostic performance was assessed by Deek’s plot using Stata version 14.0 (Stata Corp, College Station, TX, USA). An asymmetric or skewed funnel plot, *p *< 0.05 of Begg’s test or Deek’s test, was used to demonstrate the possibility of publication bias (15). The heterogeneity of included studies was evaluated by the inconsistency index (*I*^2^) and Cochran’s *Q*-tests. A random-effects model was applied in subsequent pooling when *I*^2 ^> 50% or *p *< 0.05 for Cochran’s *Q*-test (suggesting statistically significant heterogeneity); the fixed-effects model was used when *I*^2 ^< 50% (16).

### Meta-Regression and Subgroup Analysis

The Spearman correlation between the logit of sensitivity and the logit of 1−specificity was used to assess the threshold effect by Meta-DiSc version 1.4 (Universidad Complutense, Madrid, Spain); the threshold effect is one of the primary causes of heterogeneity in diagnosis-accuracy studies. A value of *p *< 0.05 for Spearman correlation analysis indicated the potential of a threshold effect. If heterogeneity resulting from the threshold effect was found, data were pooled by fitting a hierarchical summary receiver operating characteristic curve (HSROC), and the curve was pooled through the area under the receiver operating characteristic curve (AUC). Other factors may also contribute to heterogeneity in diagnosis-accuracy studies except for the threshold effect. Meta-regression of diagnostic performances was used to explore other factors (including study designs, lesion types, and machine types) that could significantly influence diagnostic values. Pooling could be performed in the homogeneous subgroup only if heterogeneity was related to other factors instead of the threshold effect. The sensitivity analysis was used to evaluate the stability and reliability of the combined results of meta-analysis and whether the combined results were significantly affected by a single study. Therefore, the sensitivity analysis was carried out by reducing one article at a time using Stata Version 14.0.

### Data Synthesis

Forest plots were used for continuous variables, and the standardized mean difference (SMD) between malignant lesions and benign lesions was calculated by RevMan Version 5.3. The diagnostic performances, including sensitivity, specificity, positive likelihood ratio (PLR), negative likelihood ratio (NLR), diagnostic odds ratio (DOR), and the area under the receiver operating characteristic curve (AUC), were pooled by a bivariate regression model using the Stata Version 14.0. The likelihood ratio and post-test probability were also significant to disease diagnosis (17), presenting the possibility that a patient was diagnosed with a certain disease using MRI parameters (18). The summary receiver operating characteristic curves (SROCs) and Fagan’s nomograms were also used to evaluate the diagnostic values and predict post-test probabilities of ADC, *D*, D*, and *f* values.

## Results

### Literature Search and Selection

**Figure 1:**
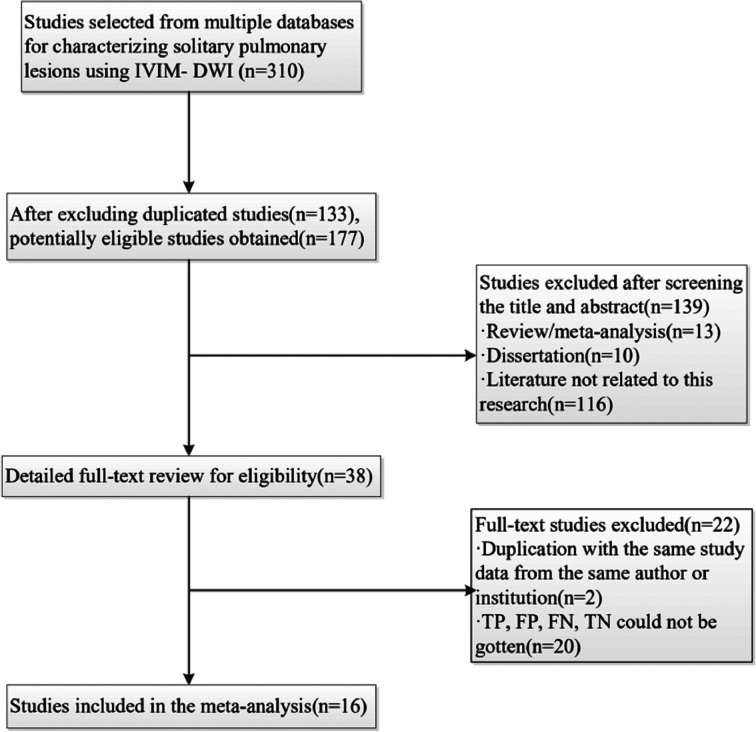
Flowchart detailing the study selection process. Sixteen studies that met the inclusion criteria were finally included. FN, false negative; FP, false positive; TN, true negative; TP, true positive.

A total of 310 studies were obtained, after which 133 duplication studies were excluded. Next, the titles and abstracts were screened, which led to exclusion of 139 additional studies (study reviews, meta-analysis, dissertations, or those where IVIM-DWI was not the main diagnosis measurement). We scanned the full texts of the remaining 38 studies in detail and excluded 22 studies for the following reasons: (a) a lack of sufficient data, (b) low-quality assessment, and (c) IVIM-DWI was applied for other purposes. Finally, 16 eligible studies (19–34) comprising 714 malignant and 355 benign lesions were included in the analysis. A flowchart of the study selection process is shown in Figure 1. The basic information and diagnostic performance of each study are presented in Tables 1 and 2, respectively.

**Table 1:** Basic information for each study.

Author	Year	Study design	Machine type	*b* values (s/mm^2^)	TR (ms)	TE (ms)	Patient	Age (years)	Lesion type
Jiang (19)	2020	PS	3.0 T Siemens	0, 50, 100, 150, 200, 250, 300, 500, 800, 1,000	7,600	67	121	60.2	N/M
Zhou (20)	2019	PS	1.5 T GE	0, 20, 50, 100, 150, 200, 400, 600, 1,000	2,500	76	64	52.8 ± 10.5	N/M
Wang (21)	2019	PS	3.0 T Philips	0, 5, 10, 15, 20, 25, 50,80, 150, 300, 500, 800, 1,000	1,111	55	50	Benign: 7.50 ± 15.74;	N/M
								Malignant: 49.00 ± 9.23	
Jiao (22)	2019	RS	3.0 T GE	0, 25, 50, 75, 100, 200, 400, 600, 800, 1,000	6,600	73	96	NA	N/M
Hong (23)	2019	PS	3.0 T Siemens	0, 50, 100, 150, 200, 250, 300, 500, 800, 1,000	7,600	67	30	59.3 ± 11.9	N
Yang (24)	2018	PS	1.5 T Siemens	0, 50, 150, 200, 400, 600, 800	4,600	86	57	58	N
Zeng (25)	2017	PS	3.0 T GE	0, 100, 200, 400,600, 1,000	899	56	168	Benign: 55.6 ± 9.5;	N/M
								Malignant: 58.9 ± 8.7	
Wan (26)	2017	RS	3.0 T Philips	0, 5, 10, 15, 20, 25, 50, 80, 150, 300, 500, 800, 1,000	1,111	55	62	56	N/M
Zhou (27)	2016	RS	1.5 T GE	20, 50, 100, 150, 200, 400, 600, 1,000	NA	NA	66	53.1	N
Yuan (28)	2016	PS	3.0 T Siemens	0, 50, 100, 150, 200, 400, 600, 800	6,800	98	81	NA	N/M
Huang (29)	2016	RS	3.0 T GE	0, 10, 25, 50, 100, 200, 400, 600, 800, 1,000	NA	64.7	45	57.4 ± 13.2	N/M
Deng (30)	2016	PS	3.0 T Philips	0, 25, 50,75, 100, 200, 400, 600, 800, 1,000	899	56	38	58.80 ± 10.93	N/M
Lei (31)	2015	PS	3.0 T Philips	0, 25, 50, 75, 100, 200, 400, 600, 800, 1,000	899	56	38	55.0 ± 12.1	N/M
Koyama (32)	2015	PS	1.5 T Philips	0, 50, 100, 150, 300, 500, 1,000	NA	70	32	68.2 ± 7.3	N
Wang (33)	2014	PS	3.0 T GE	0, 50, 100, 150, 200, 400, 600, 1,000, 1,500	12,000–14,000	70	38	Benign:55.0 ± 14.8;	N/M
								Malignant:57.7 ± 12.7	
Wang (34)	2014	PS	1.5 T Siemens	0, 5, 10, 15, 20, 25, 50, 80, 150, 300, 500, 800	2,200	70	31	2.89 ± 1.19	N/M

*PS, prospective study; RS, retrospective study; TR, repetition time; TE, echo time; NA, not available; N, nodule; N/M, nodules or masses.*

**Table 2:** Diagnostic performance for each study.

Indicator	Author	Year	Threshold	AUC	Sensitivity	Specificity	TP	FP	FN	TN
ADC	Jiang	2020	1.46	0.81	0.92	0.63	81	12	7	21
	Zhou	2019	1.57	0.71	0.91	0.59	38	9	4	13
	Wan	2017	1.32	0.83	0.86	0.82	44	2	7	9
	Deng	2016	1.02	NA	0.73	0.88	22	1	8	7
	Yuan	2016	NA	NA	0.81	0.81	39	9	9	39
	Wang	2019	1.27	0.85	0.85	0.72	25	6	5	14
	Hong	2019	1.44	0.79	0.81	0.73	17	3	4	8
	Yang	2018	NA	0.82	0.69	0.90	28	2	13	14
	Huang	2018	1.55	0.81	0.89	0.67	27	5	3	10
	Zhou	2016	1.57	0.73	0.88	0.75	41	5	6	14
	Koyama	2015	0.90	0.61	0.70	0.33	19	6	8	3
	Wang	2014	1.41	0.95	0.90	0.97	28	1	3	30
*D*	Jiang	2020	1.23	0.88	0.91	0.89	80	3	8	30
	Zhou	2019	1.25	0.73	0.95	0.55	40	10	2	12
	Wan	2017	1.20	0. 88	0.92	0.82	47	2	4	9
	Yuan	2016	NA	NA	0.91	0.39	44	30	4	18
	Wang	2019	1.19	0.89	0.89	0.75	27	5	3	15
	Yang	2018	NA	0.86	0.69	1.00	28	0	13	16
	Huang	2018	1.04	0.93	0.94	0.75	28	4	2	11
	Zhou	2016	1.25	0.71	0.95	0.56	45	8	2	11
	Jiao	2019	0.99	0.81	0.76	0.79	45	8	14	29
	Koyama	2015	0.60	0.56	0.70	0.11	19	8	8	1
	Zeng	2017	0.91	0.94	0.97	0.75	113	13	3	39
	Wang	2014	0.90	0.84	0.96	0.80	22	3	1	12
	Wang	2014	0.98	0.76	0.87	0.67	27	10	4	21
D*	Jiang	2020	15.90	0.70	0.79	0.63	70	12	18	21
	Zhou	2019	8.82	0.68	0.71	0.59	30	9	12	13
	Yuan	2016	NA	NA	0.48	0.69	23	15	25	33
	Wang	2019	7.42	0.31	0.35	0.27	11	15	20	5
	Huang	2018	17.94	0.61	0.77	0.46	23	8	7	7
	Zhou	2016	13.29	0.68	0.65	0.69	31	6	16	13
	Zeng	2017	NA	0.84	0.89	0.73	103	14	13	38
	Wang	2014	>3.70	0. 68	0.83	0.60	19	6	16	13
*f*	Deng	2016	0.37	NA	0.80	0.75	24	2	6	6
	Yuan	2016	NA	NA	0.61	0.69	29	15	19	33
	Wang	2019	0.45	0.29	0.15	0.50	5	10	26	10
	Yang	2018	NA	0.74	0.98	0.38	40	10	1	6
	Huang	2018	0.62	28.35	0.75	0.43	23	9	8	6
	Zhou	2016	0.40	0.67	0.73	0.56	34	8	13	11
	Lei	2015	0.38	0.83	0.8	0.75	24	2	6	6
	Koyama	2015	0.15	0.64	0.78	0.22	21	7	6	2
	Zeng	2017	NA	0.76	0.47	0.94	54	3	62	49
	Wang	2014	≤39. 3%	0.64	0.52	0.80	12	3	11	12
	Wang	2014	24.93%	0.76	0.81	0.55	25	14	6	17

NA, not available; ADC, apparent diffusion coefficient; D, tissue diffusivity; D*, *pseudo-diffusivity; f, perfusion fraction; AUC, Area under the curve; TP, true positive; FP, false positive; FN, false negative; TN, true negative.*

### Quality Assessment

**Figure 2:**
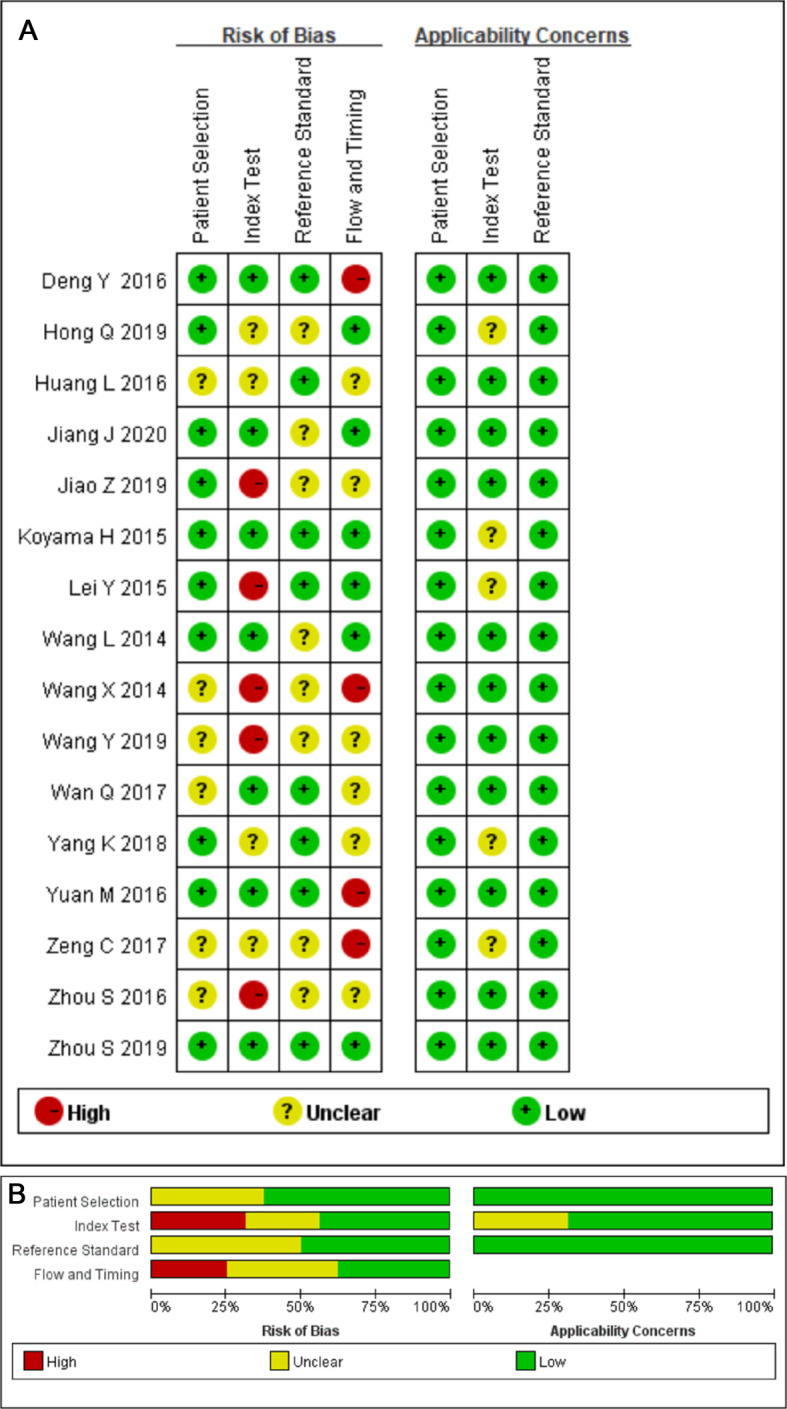
(**A**) Methodological quality summary. (**B**) Methodological quality graph.

The outcome of the QUADAS 2 assessment is shown in Figure 2. The overall quality of included studies was acceptable. Six studies were marked as “unclear” since their patient selection method was unclear. In the index test domain, six studies were marked as “unclear or “high risk of bias” because of the uncertainty concerning the process of interpreting result, and four studies because of the uncertainty concerning whether a threshold was prespecified or not while being used. Applicability of the index test showed unclear concern because threshold values of some parameters were missing (*n* = 5). Eight studies were marked as “unclear” in the reference standard domain since the application of the blind method while interpreting the gold standard result was unclear. In the flow and timing domain, 10 studies were marked as “unclear” or “high risk of bias” due to ambiguity related to the existence of an appropriate time interval between the index test and the reference standard; four of these ten studies were marked as high risk of bias, three (25, 30, 33) due to inconsistent application of the reference standard and one (28) due to the fact that four patients were excluded from statistical analysis.

### Publication Bias and Heterogeneity Analysis

**Figure 3:**
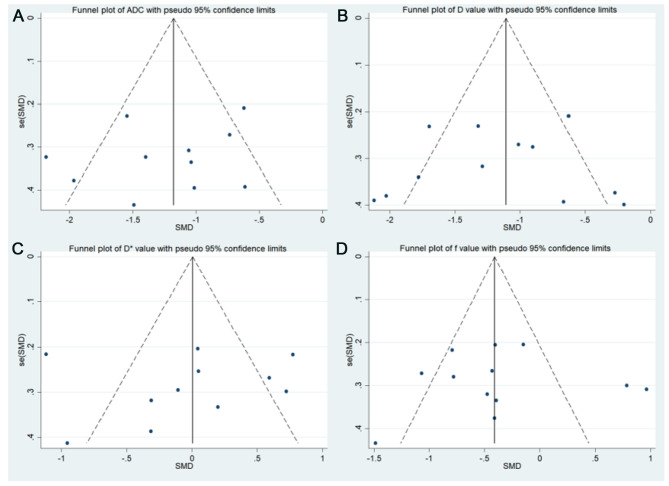
Funnel plots. (**A**) Apparent diffusion coefficient (ADC value); (**B**) tissue diffusivity (*D* value); (**C**) pseudo-diffusivity (D* value); and (**D**) perfusion fraction (*f* value).

Funnel plots of ADC, *D*, D*, and *f* values are shown in Figure 3. All funnel plots were symmetrical, and both Begg’s test (*p *= 0.592, 0.542, 0.350, and 0.464 for ADC, *D*, D*, and *f* values, respectively) and Egger’s test (*p *= 0.370, 0.830, 0.759, and 0.617 for ADC, *D*, D*, and *f* values, respectively) showed no positive results, suggesting no obvious publication bias in ADC, *D*, D*, and *f* values.

In the heterogeneity analysis, Cochran’s *Q*-test suggested moderate heterogeneity in ADC (*I*^2 ^= 66% and *p *= 0.001 < 0.05) and high heterogeneity in the *D* value (*I^2 ^*= 74% and *p *< 0.001), D* value (*I*^2 ^= 82% and *p *< 0.001), and *f* value (*I*^2 ^= 79% and *p *< 0.001). Threshold effect analysis was performed by a Spearman rank correlation test and determined to be 0.252 (*p *= 0.429), 0.132 (*p *= 0.667), −0.252 (*p *= 0.548), and 0.370 (*p *= 0.263) for ADC, *D*, D*, and *f* values, respectively, indicating no threshold effect causing the variations in the diagnosis accuracy estimates.

### Quantitative Analysis

#### Differential Diagnosis of Solitary Pulmonary Nodules and Masses by ADC

**Figure 4:**
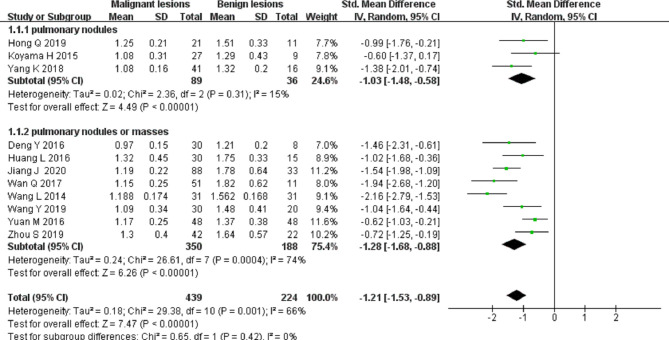
Forest plot of the mean value of the apparent diffusion coefficient (ADC) between malignant and benign pulmonary nodules and masses.

Eleven studies on ADC applied in differentiating solitary pulmonary nodules and masses were included in the analysis. The forest plot in Figure 4 presents the distribution of ADC between the malignant and the benign. An SMD of −1.21 [95% CI, −1.53, −0.89] (*p < *0.001) between the malignant and the benign was calculated by a random-effects model.

#### Differential Diagnosis of Solitary Pulmonary Nodules and Masses by the *D* Value

**Figure 5:**
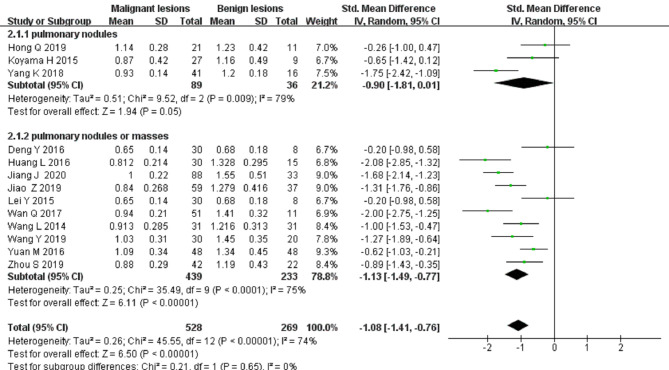
Forest plot of the mean value of the tissue diffusivity (*D* value) between malignant and benign pulmonary nodules and masses.

Thirteen studies on the *D* value used to distinguish solitary pulmonary nodules and masses were included for analysis. The forest plot in Figure 5 presents the distribution of the *D* value between the malignant and benign lesions. An SMD of −1.08 [95% CI, −1.41, −0.76] (*p < *0.001) between the malignant and benign lesions was calculated by a random-effects model.

#### Differential Diagnosis of Solitary Pulmonary Nodules and Masses by the D* Value

**Figure 6:**
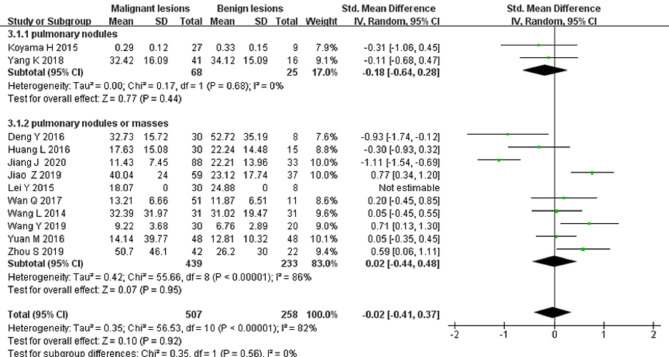
Forest plot of the mean value of the pseudo diffusivity (D* value) between malignant and benign pulmonary nodules and masses.

Twelve studies on the D* value used to differentiate solitary pulmonary nodules and masses were included for analysis. The forest plot in Figure 6 presents the distribution of the D* value between the malignant and benign nodules and masses. An SMD of −0.02 [95% CI, −0.41, 0.37] (*p > *0.05) between the malignant and benign nodules and masses was calculated by a random-effects model.

#### Differential Diagnosis of Solitary Pulmonary Nodules and Masses by the *f* Value

**Figure 7:**
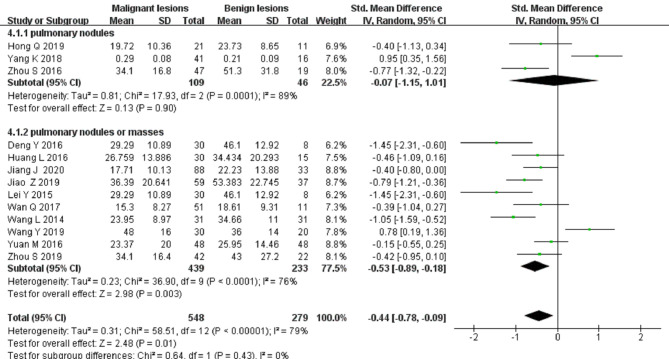
Forest plot of the mean value of the perfusion fraction (*f* value) between malignant and benign pulmonary nodules and masses.

Thirteen studies on the *f* value used to differentiate solitary pulmonary nodules and masses were included for analysis. The forest plot in Figure 7 presents the distribution of the *f* value between the malignant and benign lesions. An SMD of −0.44 [95% CI, −0.7, −0.09] (*p < *0.05) between the malignant and benign lesions was calculated by a random-effects model.

### Meta-Regression and Subgroup Analysis

There was no statistical difference concerning the influence of study designs, machine types, and lesion types on pooled ADC, *D*, D*, and *f* values (all *P*’s* *> 0.05). There was a significant difference concerning study design in the *D* value (*p *= 0.04). The SMD of the *D* value between malignant and benign lesions of the prospective study (−0.96 [95% CI, −1.14, −0.78]) was significantly larger than that of the retrospective study (−1.63 [95% CI, −1.98, −1.29]).

We then explored the potential factors (apart from the threshold effect) that caused the heterogeneity of ADC, *D*, D*, and *f* values with meta-regression analysis (Table 3**)**. For ADC, the pooled sensitivity of study designs (“prospective design” vs. “retrospective design”) (*p *< 0.001), lesion types (“nodules” vs. “nodules or masses”) (*p *< 0.001), and machine types (“3.0 T” vs. “1.5 T”) (*p < *0.01) was statistically significant. For the *D* value, pooled sensitivity of lesion types (*p *< 0.001) was statistically significant. For the *f* value, the pooled sensitivity of machine types was statistically significant (*p *< 0.05).

**Table 3:** Results of multiple univariate meta-regression and subgroup analysis.

Parameter	Category	Number of study	Sensitivity (95% CI)	*p*	Specificity (95% CI)	*p*
ADC	PS	9	0.83 (0.77, 0.88)	0.00	0.76 (0.66, 0.86)	0.44
	RS	3	0.88 (0.81, 0.95)		0.75 (0.56, 0.93)	
	N	4	0.78 (0.70, 0.86)	0.00	0.71 (0.54, 0.88)	0.13
	N/M	8	0.87 (0.83, 0.91)		0.77 (0.68, 0.87)	
	3.0 T	7	0.85 (0.80, 0.91)	0.00	0.75 (0.64, 0.87)	0.21
	1.5 T	5	0.82 (0.75, 0.90)		0.76 (0.63, 0.89)	
*D*	PS	9	0.90 (0.85, 0.96)	0.06	0.70 (0.55, 0.85)	0.46
	RS	4	0.91 (0.83, 0.98)		0.74 (0.53, 0.95)	
	N	3	0.81 (0.68, 0.94)	0.00	0.64 (0.34, 0.94)	0.44
	N/M	10	0.92 (0.88, 0.96)		0.73 (0.60, 0.86)	
	3.0 T	8	0.92 (0.87, 0.97)	0.21	0.76 (0.62, 0.89)	0.81
	1.5 T	5	0.86 (0.77, 0.95)		0.63 (0.41, 0.85)	
D*	PS	6	0.66 (0.51, 0.81)	0.50	0.61 (0.51, 0.72)	0.82
	RS	2	0.71 (0.47, 0.95)		0.59 (0.37, 0.81)	
	N	1	0.66 (0.30, 1.00)	0.88	0.69 (0.41, 0.96)	0.85
	N/M	7	0.67 (0.54, 0.81)		0.60 (0.50, 0.71)	
	3.0 T	6	0.67 (0.52, 0.81)	0.62	0.60 (0.49, 0.72)	0.49
	1.5 T	2	0.69 (0.45, 0.93)		0.64 (0.44, 0.84)	
*f*	PS	9	0.70 (0.54, 0.85)	0.72	0.65(0.50, 0.80)	0.51
	RS	2	0.74 (0.44, 1.00)		0.49 (0.16, 0.83)	
	N	3	0.86 (0.72, 1.00)	0.28	0.40 (0.15, 0.65)	0.08
	N/M	8	0.62 (0.46, 0.78)		0.70 (0.57, 0.83)	
	3.0 T	7	0.59 (0.43, 0.76)	0.01	0.72 (0.59, 0.85)	0.18
	1.5 T	4	0.85 (0.72, 0.97)		0.45 (0.24, 0.66)	

*PS, prospective study; RS, retrospective study; N, nodule; N/M, nodules or masses*.

### Sensitivity Analysis

Table 4 shows the combined DOR and 95% CI calculated by eliminating a study at a time. Regardless of which study was eliminated, the combined DOR did not significantly change, indicating that the result of this analysis was not excessively dependent on one certain study and that the conclusion was stable.

**Table 4:** Influence of each study on the outcome of the meta-analysis.

	Study	DOR	(95% Cl)	
ADC	Jiang	2.77	2.28	3.37
	Zhou	2.81	2.32	3.41
	Wan	2.93	2.40	3.58
	Deng	2.97	2.43	3.63
	Yuan	2.70	2.21	3.29
	Wang	2.84	2.34	3.44
	Hong	2.87	2.36	3.48
	Yang	3.00	2.44	3.69
	Huang	2.81	2.32	3.41
	Zhou	2.84	2.33	3.45
	Koyama	3.08	2.52	3.77
	Wang	2.62	2.16	3.17
	**Combined**	2.85	2.36	3.44
*D* value	Jiang	3.68	2.91	4.65
	Zhou	3.73	2.99	4.65
	Wan	3.85	3.07	4.82
	Yuan	3.83	3.06	4.80
	Wang	3.74	2.99	4.68
	Yang	4.08	3.20	5.20
	Huang	3.73	2.99	4.66
	Zhou	3.73	2.99	4.65
	Jiao	4.05	3.17	5.17
	Koyama	4.27	3.37	5.41
	Zeng	3.32	2.67	4.13
	Wang	3.68	2.95	4.58
	**Combined**	3.79	3.05	4.72
D* value	Jiang	1.62	1.37	1.91
	Zhou	1.66	1.42	1.96
	Yuan	1.70	1.44	2.00
	Wang	1.85	1.57	2.18
	Huang	1.67	1.43	1.96
	Zhou	1.68	1.42	1.98
	Zeng	1.39	1.19	1.62
	Wang	1.69	1.44	1.99
	**Combined**	1.66	1.42	1.93
*f* value	Deng	1.56	1.36	1.80
	Yuan	1.55	1.35	1.79
	Wang	1.72	1.49	1.96
	Yang	1.53	1.34	1.75
	Zhou	1.59	1.38	1.84
	Lei	1.56	1.36	1.80
	Koyama	1.62	1.41	1.86
	Zeng	1.53	1.29	1.83
	Wang	1.57	1.37	1.81
	Wang	1.53	1.34	1.76
	**Combined**	1.58	1.38	1.81

ADC, apparent diffusion coefficient; D, tissue diffusivity; D*, *pseudo diffusivity; f, perfusion fraction.*

### Diagnostic Performance

**Figure 8:**
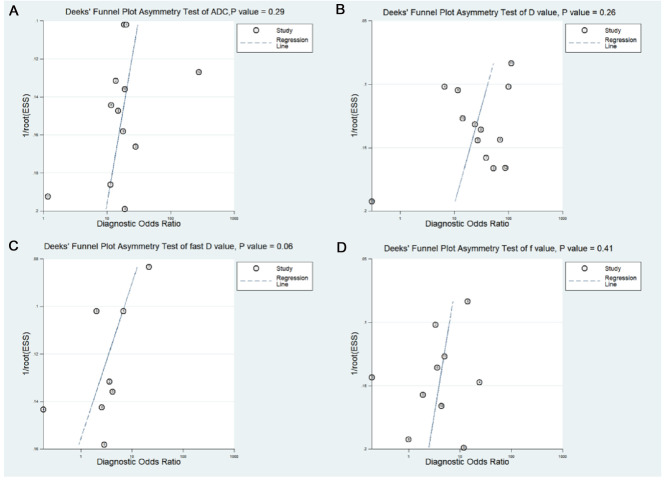
Deeks’ funnel plots regarding the diagnostic performance for the (**A**) apparent diffusion coefficient (ADC value); (**B**) tissue diffusivity (*D* value); (**C**) pseudo-diffusivity (D^*^ value); and (**D**) perfusion fraction (*f* value).

**Figure 9:**
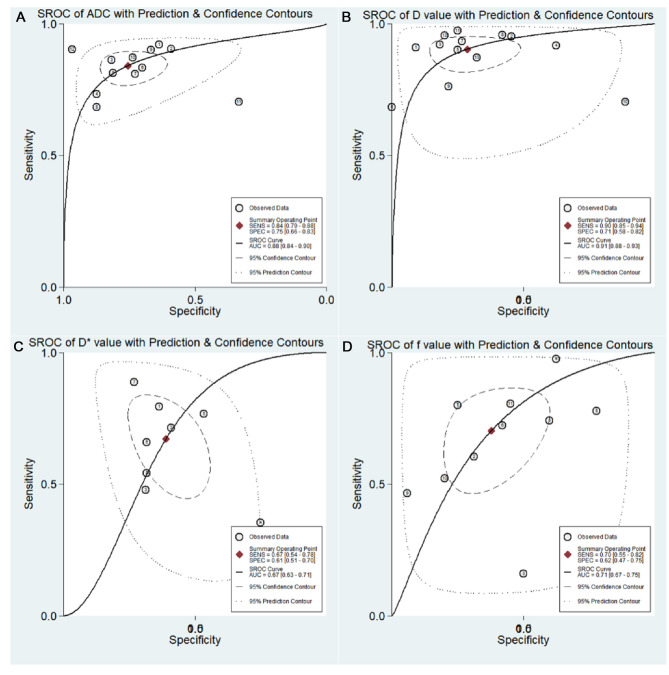
Summary receiver operating characteristic (SROC) curve of the (**A**) apparent diffusion coefficient (ADC value); (**B**) tissue diffusivity (*D* value); (**C**) pseudo-diffusivity (D* value); and (**D**) perfusion fraction (*f* value).

The results of pooled analysis of ADC, *D*, D*, and *f* values are shown in Table 5. Deeks’ funnel plots (Figure 8) suggested no obvious publication bias in ADC, *D*, D*, and *f* values (*p *= 0.29, 0.26, 0.06, and 0.41, respectively). Figure 9 shows the summary receiver operating characteristic curves of ADC, *D*, D*, and *f* values. The *D* value showed the best diagnostic performance (sensitivity = 0.90; specificity = 0.71; AUC = 0.91), followed by ADC (sensitivity = 0.84; specificity = 0.75; AUC = 0.88), *f* value (sensitivity = 0.70; specificity = 0.62; AUC = 0.71), and D^*^ value (sensitivity = 0.67; specificity = 0.61; AUC = 0.67).

**Table 5:** Pooled estimates and heterogeneity measures for ADC, *D*, D*, and *f* value.

Index	Sensitivity	Specificity	PLR	NLR	DOR	AUC	*I*^2^ (%)	
							Sensitivity	Specificity
ADC	0.84 (0.79, 0.88)	0.75 (0.66, 0.83)	3.4 (2.4, 4.9)	0.21 (0.16, 0.28)	16 (9, 28)	0.88 (0.84, 0.90)	50.67%	56.05%
*D*	0.90 (0.85, 0.94)	0.71 (0.58, 0.82)	3.2 (2.1, 4.9)	0.14 (0.08, 0.22)	23 (11, 50)	0.91 (0.88, 0.93)	77.66%	79.45%
D*	0.67 (0.54, 0.78)	0.61 (0.51, 0.70)	1.7 (1.2, 2.5)	0.54 (0.33, 0.87)	3 (1, 8)	0.67 (0.63, 0.71)	87.71%	60.38%
*f*	0.70 (0.55, 0.82)	0.62 (0.47, 0.75)	1.9 (1.3, 2.7)	0.48 (0.30, 0.75)	4 (2, 8)	0.71 (0.67, 0.75)	88.17%	77.70%

ADC, apparent diffusion coefficient; D, tissue diffusivity; D*, pseudo-diffusivity; f, perfusion fraction; PLR, positive likelihood ratio; NLR, negative likelihood ratio; DOR, diagnostic odds ratio; AUC, area under the curve; I^2^, *inconsistency index.*

### Post-Test Probabilities

**Figure 10:**
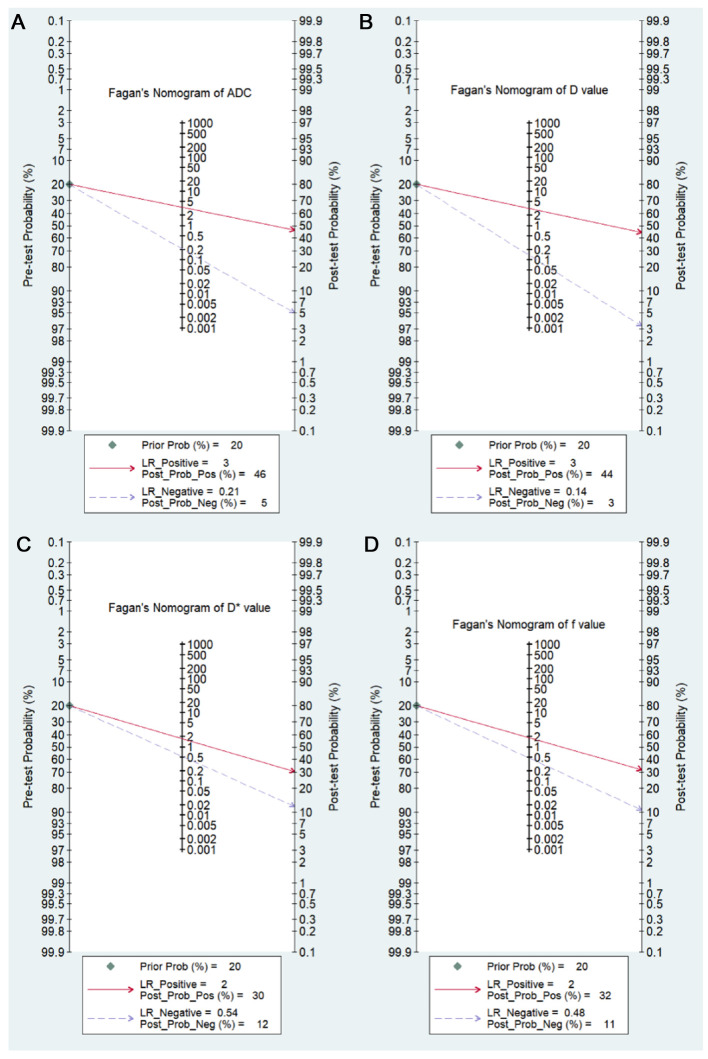
Fagan’s nomogram of the (**A**) apparent diffusion coefficient (ADC value); (**B**) tissue diffusivity (*D* value); (**C**) pseudo-diffusivity (D* value); and (**D**) perfusion fraction (*f* value).

Fagan’s nomograms of ADC, *D*, D*, and *f* values were used to predict post-test probabilities (Figure 10). All the pretest probabilities were set to 20% by default. Lower ADC and *D* values corresponded to a positive event associated with diagnosis of malignant pulmonary nodules and masses. An adverse event associated with benign nodules and masses corresponded to higher ADC and *D* values. The post-test probability increased to 46% with a PLR of 3.0 and decreased to 5% with an NLR of 0.21. Therefore, the diagnostic preference for malignant pulmonary lesions improved when using the ADC (a lower ADC). In contrast, when an adverse event occurred (a higher ADC), the probability of diagnosing malignant pulmonary lesions considerately decreased to 5%. Likewise, the post-test probability for a positive issue was 44% with a PLR of 3.0 and plunged to 3% with an NLR of 0.14 using the *D* value. The post-test probability of a positive issue was 30% with a PLR of 2.0 and decreased to 12% with an NLR of 0.54 by using the D* value. The post-test probability of a positive issue was 32% with a PLR of 2.0 and decreased to 11% with an NLR of 0.48 by using the *f* value. These data suggested that both ADC and IVIM parameters were useful in improving the accuracy of differentiating benign and malignant pulmonary nodules and masses.

## Discussion

This meta-analysis assessed the diagnostic performance of IVIM-DWI for the differential diagnosis of solitary pulmonary masses and nodules. The pooled results suggested that the *D* value of IVIM-DWI had better diagnostic performance compared with the monoexponential ADC value.

Various aspects were assessed, including the threshold effect, meta-regression, subgroup analysis, and sensitivity analysis. Thus, the analysis and the outcomes were more precise and convincing.

The SMDs of ADC, *D*, and *f* values in malignant lesions were lower than those in benign lesions with statistical significance. The ADC value quantitatively expresses the diffusion characteristic of tissues; the ADC value is associated with tissue cellularity, cell density, and extracellular–intracellular components. A lower ADC value of malignant tissue usually results from the microstructural environment with dense cell membranes, larger cell nucleus, and higher cellular density acting as a diffusion barrier that characterizes the malignant lesion (35).

The *D* value, which represents the pure diffusion coefficient, negatively correlates with tumor cellularity (36). The D* value is proportionate to the blood velocity and capillary segment length in IVIM theory (13). The increased D* value may result from the angiogenesis of immature vessels in lung cancer, leading to larger blood flow velocity and capillary segment length in lung cancer (20). Since increasing angiogenesis is also a characteristic of benign lesions, higher D* may also be seen in benign lesions (37). The *f* value primarily reflects the proportion of blood perfusion in the whole diffusion movement of the tumor and could represent the percentage of capillary capacity in the voxel range to the whole tissue volume (38). Previous studies suggested a higher *f* value in benign lesions compared to that in malignant lesions (20, 30). This may be because many benign lesions are seen as inflammatory granulomas or sclerosing hemangiomas with hypervascular features. However, the relaxation effects and the T2 may also be another potential cause affecting the *f* value. In addition, previous studies have suggested that the *f* value has no significant characteristics in differentiating lung cancer from benign lesions (20, 28). The homogeneity test showed moderate or high heterogeneity with reference to the sensitivity or specificity of each parameter. In this case, it was not enough to just pool sensitivity or specificity, but it was essential to explore the sources of heterogeneity (including threshold effect) in a meta-analysis. Thus, the sources of heterogeneity were investigated in this meta-analysis.

In this study, no threshold effect was found in the analysis by the Spearman correlation coefficient, suggesting that there might be other sources that cause the heterogeneity. Thus, we explored the potential factors regarding study designs, lesion types, and machine types in the meta-regression analysis. The statistical significance was found in the pooled sensitivity of study designs (“retrospective study” vs. “prospective study”), lesion types (“nodules” vs. “nodules or masses”), and machine types (“3.0 T” vs. “1.5 T”), concerning the ADC value, in the pooled sensitivity of lesion types concerning the *D* value, and in the pooled sensitivity of machine types concerning the *f* value. This indicates that these factors may result in heterogeneity. Furthermore, in the subgroup analysis of the ADC value, significant differences were found in the sensitivity of study designs, lesion types, and machine types, suggesting that ADC had higher sensitivity in “retrospective study” than that in “prospective study,” in “nodules or masses” than that in “nodules,” and in “3.0 T” than that in the “1.5 T” MRI scanner. In the subgroup analysis of the *D* value, the statistically significant difference was found in the sensitivity of lesion types, suggesting a higher sensitivity in “nodules and masses” compared to that in “nodules.” In the subgroup analysis of the *f* value, the significant difference was found in the sensitivity of the machine type, suggesting that the *f* value had higher sensitivity in “1.5 T” than that in the “3.0 T” MRI scanner. No significant change was found in the combined DOR while excluding any one of these studies, indicating the results of our meta-analysis were generally stable and reliable.

The study design was likely to cause heterogeneity since bias and confounding are more common in “retrospective studies” than in “prospective studies” (39). “Nodules or masses” showed higher sensitivity in both ADC and *D* values compared to “nodules.” Pulmonary nodules are defined as focal opacities that measure up to 3 cm in diameter, while pulmonary masses are ≥3 cm in diameter. Regier et al. (40) found the sensitivity of DWI was only 43.8% for nodules ≤5 mm in diameter and increased to 86.4% for larger diameter (6–9 mm) and 97% for nodules ≥10 mm. Moreover, Jiang and colleagues (19) assumed that various factors regarding motion, vulnerability artifacts, and the partial volume effect had an obvious impact on smaller lung lesions. Jiang et al. (41) suggested that the nodule with a diameter smaller than 2 cm or a lower lung zone location would negatively affect the reproducibility. In addition, Koo et al. (42) found that most nodules (74%) in their study were <2 cm, and nearly half of the lesions were in the lower lobes. The “3.0 T” MRI scanner showed a higher sensitivity for ADC than the “1.5 T” MRI scanner. In contrast, the sensitivity for the *f* value was higher in the “1.5 T”. Ohba and his team (43) indicated that both the “1.5 T” and “3.0 T” MRI scanners showed similar performance in assessing malignant pulmonary nodules. Schmidt et al. (44) indicated that 1.5 T MRI is the preferred imaging modality in a comparative study of high-resolution whole-body MRI applications at 1.5 T and 3.0 T.

Apart from the aspects mentioned above, the influence of the *b* values on the heterogeneity could not be ignored. The number of *b* values was viewed to improve the separation of diffusion and perfusion (45). Additionally, lower *b* values were important in gaining perfusion-sensitive information (46). At the same time, the number and range of *b* values used in published studies substantially varied, revealing an obvious lack of consensus. Therefore, it is essential to reach a consensus on the number and range of *b* values in future research. Moreover, advanced MRI technologies, such as the MRI respiratory triggering technology and advanced navigation platform, which could overcome challenges of the movement and breathing artifacts as well as the susceptibility artifacts caused by the interfaces between different tissues and the overall low proton density of the lung, should be applied.

This study has a few limitations. First, this meta-analysis was only based on published studies, which might have led to overestimating the true effect. Second, since there are limited numbers of publications that included patients with solitary pulmonary nodules, we were unable to analyze the diagnostic performance of IVIM-DWI from the perspective of various sizes of nodules. Third, although meta-regression analysis suggested various aspects attributing to the heterogeneity, it was still not enough to explore heterogeneity through the analysis due to the differences in the scanning method and acquisition protocol. However, the variants such as *b* values, cutoff values, repetition time (TR), and echo time (TE) had too many included variables, which resulted in the difficulty in conducting subgroup analysis.

## Conclusion

Overall, the pooled results suggested that the IVIM-DWI could be a valuable technique for the analysis of pulmonary nodules and masses. This meta-analysis first explored the heterogeneity of the lesion types concerning nodules and masses. The diagnostic performance shown in a subgroup analysis of the studies with masses or nodules is superior to the studies that only reported on nodules. Since MRI scanner hardware and sequence developments have achieved notable progress, IVIM-DWI might become an alternative diagnostic technique for malignant and benign differentiation of pulmonary masses and nodules.

## Data Availability

The original contributions presented in the study are included in the article/Supplementary Material, further inquiries can be directed to the corresponding author/s.
